# Pre-operative embolization and excision of vagal schwannoma with rich vascular supply: A case report and literature review

**DOI:** 10.1097/MD.0000000000028760

**Published:** 2022-01-28

**Authors:** Tao Xu, Yue Liu, Siyuan Li, Lei Zheng

**Affiliations:** Department of Oral and Maxillofacial of Peking University International Hospital, China.

**Keywords:** blood supplied, embolization, vagal schwannoma

## Abstract

**Rationale:**

Schwannomas are benign tumors wrapped in the nerve sheath and can originate from the myelin sheath of the cranial nerve. In previous literature reports, most of the tumors were solid tumors, which can be removed only by surgery. This case report describes a unique case of vagal schwannoma, which, unlike previous cases, involves a dominant arterial supply, and discusses the pre-operative evaluation, relevant radiographic findings, and surgical process of the case in detail.

**Patient concerns:**

A 31-year-old woman sought treatment for pain in the left side of the neck when turning her head. A mass on the left side of the neck was found on enhanced computed tomography with a maximum diameter of 6.8 cm, along with multiple tortuous, thickened vascular shadows, and pressure on the left pharyngeal cavity.

**Diagnosis:**

The pathological results showed schwannoma.

**Interventions:**

Considering the unusual size of the tumor and thickened blood vessels revealed by pre-operative computed tomography, general anesthesia and biopsy were conducted first to confirm the diagnosis. Excessive bleeding occurred during the process; thus, the tumor was only partially removed before the wound closed after hemostasis. Digital subtraction angiography indicated that the tumor was supplied by multiple arteries, and the tumor was removed by pre-operative embolization plus intra-operative removal.

**Outcomes:**

Combined with embolization and surgical resection, the tumor was completely removed, the nerve was partially preserved, and the patient had postoperative hoarseness.

**Lesson:**

The present case indicates the possibility of vascularly supplied vagal schwannoma; thus, it is necessary to conduct pre-operative digital subtraction angiography.

## 1 Introduction

Schwannomas, first reported by Stout in 1935,^[1,2]^ are the most common type of benign tumor arising from neurocytes, namely Schwann cells. Schwannomas, constituting 5% of soft tissue tumors, usually occur in the age group of 30 to 40 years, with no gender difference.^[3,4]^ Among schwannomas that are commonly detected in the head and neck region, approximately 25% to 45% are extracranial.^[5]^ Extracranial schwannomas, which often originate from the sympathetic, vagal, and hypoglossal nerves, mostly occur in the parapharyngeal space.^[6]^ Clinically, schwannomas often manifest as painless growing masses with a relatively slow growth rate of 2.5 to 3 mm per year.^[7,8]^ After the tumor grows to a certain size that could exert compression on the surrounding tissues, clinical symptoms, such as hoarseness, coughing, dysphagia, dysphonia, may occur.^[9]^

Schwannomas can be treated via surgical methods, including total tumor resection and subtotal tumor resection (intracapsular resection). However, owing to the increasing awareness of neuroprotection and low rate of recurrence postsurgery, subtotal tumor resection has attracted increasing attention.^[10,11]^ Vocal hoarseness was once considered an inevitable complication in patients with schwannomas.^[9]^ However, recent studies have indicated that understanding the eccentric growth pattern of the tumor^[12]^ and the anatomical characteristics of the recurrent laryngeal and non-laryngeal reflex can guide surgeons to protect the nerves as much as possible to maintain good vocal function after surgery.^[13]^ In the following sections, we report a case of vascular schwannoma in which pre-operative embolization and intracapsular removal were performed to preserve most of the nervous function.

## 2 Case report

**Figure 1:**
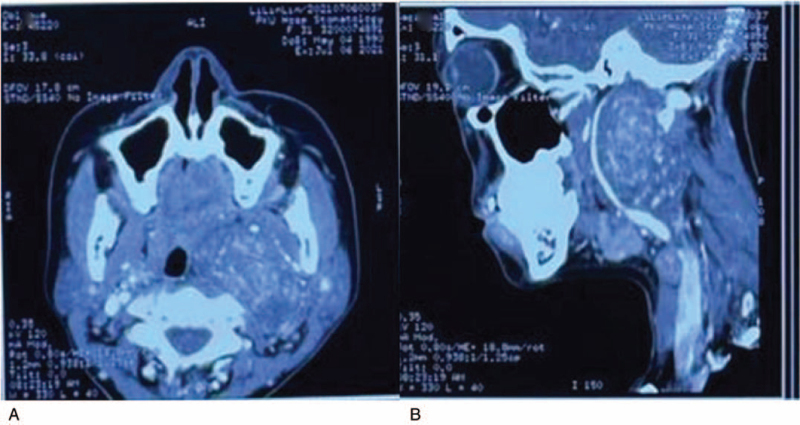
(A,B) CT showed shadows of a 6.8 × 5.4 × 3.4 cm soft tissue masses with uneven reinforcement density in the left neck and parapharyngeal area. The superior boundary of the shadows reached the skull base, the inferior boundary reached the level of the hyoid bone, and the boundaries were clear. There were speckled calcifications and thickened and tortuous blood vessels in the image. The left pharyngeal cavity was compressed and narrowed. CT = computed tomography.

A 31-year-old Chinese woman reported pain in the left side of the neck when turning and numbness of the skin on the left side of the auriculotemporal. Clinical examination revealed swelling on the left side of the neck and pharynx, and a huge mass with a hard texture was detected. Hoarseness was not observed. Enhanced computed tomography (CT) showed shadows of a 6.8 × 5.4 × 3.4 cm soft tissue masses with uneven reinforcement density in the left neck and parapharyngeal area. The superior boundary of the shadows reached the skull base, the inferior boundary reached the level of the hyoid bone and the boundaries were clear. Speckled calcifications and thickened and tortuous blood vessels were observed in the image. The left pharyngeal cavity is compressed and narrowed. Compression and deformation of the left pterygoid muscle were also observed. The internal carotid artery was internally displaced under pressure, the external carotid artery was deformed under pressure, and the internal jugular vein was externally displaced (Fig. 1).

Due to the large size of the tumor and its uncertain nature, general anesthesia and biopsy were performed first.^[14]^ During the surgery, a conventional submandibular incision was made. After the platysma was elevated, the tumor was found, and a part of the tumor was removed and sent for pathological biopsy. At the same time, it was found that there was more bleeding in the operative area than usual. After compression and suturing to stop bleeding, the wound was closed. Postoperative pathological results supported the diagnosis of schwannoma. After the surgery, the patient started to show symptoms, including hoarseness and coughing. Although the recurrent laryngeal nerve was not touched during the surgery, such symptoms could be attributed to intra-operative blood pressure injury. Postoperative otolaryngological department endoscopy revealed paralysis of the left vocal cord in the midline position. The arytenoid cartilage was positioned symmetrically on both sides.

**Figure 2:**
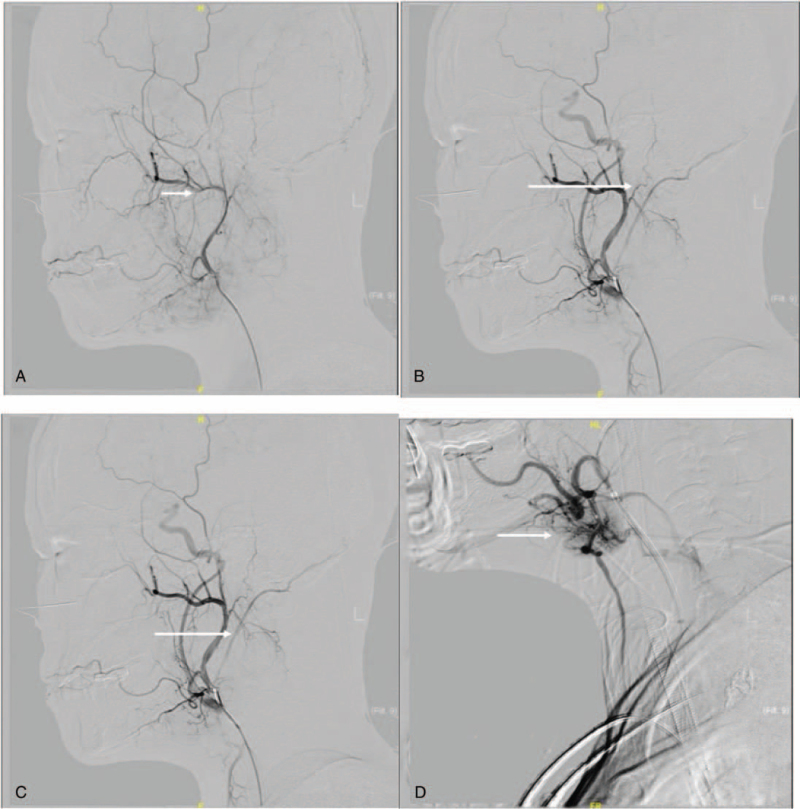
DSA showed the branches of the maxillary artery (A), the branches of the posterior auricular artery (B), and the branches of the occipital artery (C) were all involved in the blood supply of the tumor area. At the same time, a deformity vascular nest, mainly supplied by the branches of the facial artery (D), could be seen in the submandibular area. DSA = digital subtraction angiography.

While waiting for pathological results, digital subtraction angiography (DSA) was conducted.^[15]^ After inserting the contrast agent through femoral artery puncture, it was found that the branches of the maxillary artery, occipital artery, and posterior auricular artery were all involved in blood supply to the tumor area. At the same time, a deformed vascular nest, mainly supplied by the branches of the facial artery, could be seen in the submandibular area. Considering the characteristics of schwannoma and abundant blood supply, the surgical method of pre-operative embolization plus intra-operative resection was adopted (Fig. 2).

The femoral artery approach is routinely used for embolization, in combination with vascular surgery. The maxillary artery diverged 1.5 cm from the external carotid artery and then branched into the tumor, while the posterior auricular artery diverged 0.5 cm from the external carotid artery and then branched into the tumor. Therefore, glue embolization was performed at the fusion interchange between the maxillary and posterior auricular arteries. The occipital artery and branch of the facial artery that supplied the submandibular vascular nest were also embolized with glue. After embolization, the vasculature in the tumor area was significantly reduced.

After embolization was completed by an interventional vascular surgeon, the left submandibular area incision combined with the left maxillary vestibular sulcus was chosen as the surgical approach,^[16]^ extending the original biopsy incision forward to the midline and backward to the mastoid.

After incision of the subcutaneous tissue and platysma of the skin, the tissue became more edematous. Subsequently, the submandibular gland capsule was separated, part of the submandibular gland attachment was removed, and the submandibular gland was dragged forward and downward. After the carotid sheath on the deep surface of the sternocleidomastoid muscle was exposed, it was opened, and the common carotid artery, internal carotid artery, and vagal nerve were exposed, uncovering the lower edge of the tumor. The tumor was closely attached to the vagal nerve, and part of the tumor originated from the lateral wall of the common carotid artery. Thus, embolization was performed, but diffusion oozing still occurred in the tumor area. The tissues around the tumor were carefully separated, and bifurcation of the common carotid artery was detected. The internal and external carotid arteries were identified correspondingly, and the internal carotid artery was found to be compressed and displaced to the inside. Combined with the pre-operative DSA, which confirmed that the internal carotid artery was not involved in the blood supply to the tumor area, it was concluded that the tumor mainly originated from the vagal nerve and was closely related to the external carotid artery.

Based on the confirmed diagnosis, the tumor capsule was carefully separated and the blood supply arteries, to which the tumor was closely attached, were ligated. The vagal nerve trunk along the vagal nerve and upper and lower poles of the tumor were dissected. Nerve fibers were scattered on the superficial surface of the tumor and mixed into the tumor capsule. The tumor from the vagal nerve was separated and the tumor capsule adhering to the vagal nerve was partly retained. No nerve trunk passed into the tumor parenchyma. Through the space between the external pterygoid muscle and parapharyngeal space, the tumor was gradually removed, and the lateral wall of the tumor attached to the vagal nerve and part of the tumor tissue attached to the common carotid artery were retained. The common carotid artery, internal carotid artery, external carotid artery, and vagal nerve were intact, whereas the internal jugular vein was displaced anterolaterally and severely compressed.

After achieving hemostasis, the subcutaneous tissue and skin were sutured, and a negative pressure drainage tube was inserted into the wound. The patient recovered well after surgery. The drainage tube was withdrawn when the drainage volume decreased significantly on the 5th day after surgery. On the 6th day after the operation, the patient's condition of hoarseness improved, and her speech was clearer than before. On the 7th day after the operation, the sutures in the wound area were removed. Postoperative pathology showed schwannoma with a tumor size of approximately 5 × 4 × 3.5 cm. S-100 (+), SOX10 (+), CD34 (vascular +), SMA (–), KI-67 (2%).

The study was performed in accordance with the Declaration of Helsinki and International Conference on Harmonization Good Clinical Practice guidelines. The patient provided written informed consent before publication of the paper.

## 3 Discussion

### 3.1 Literature review

Schwannomas are benign tumors wrapped in the nerve sheath, which can originate from the myelin sheath of the cranial, peripheral, and autonomic nerves, but not from the olfactory and optic nerves, which have no nerve sheath.^[5]^ Schwannomas that occur in the parapharyngeal space mainly originate from the vagal, sympathetic, or hypoglossal nerves.^[6]^ They develop slowly and are typically painless. They can move along the long axis of the nerve, without moving sideways.^[17]^ Clinically, schwannomas and neurofibromas are often misdiagnosed. The main difference between the two is that schwannomas are eccentric to the originating nerve, whereas neurofibromas are spindle-shaped and located in the center of the perineurium.^[12]^ Therefore when the schwannoma is large, it will gradually compress the fibers of the parent nerve and cause nerve ischemia, resulting in corresponding clinical symptoms.

When a parapharyngeal mass is suspected to be a neurogenic tumor and the patient has clinically corresponding neurological symptoms, imaging examination is essential.^[7]^ CT examination can better show the size and shape of the tumor. The tumors were mostly round in shape with clear boundaries, accompanied by different degrees of enhancement. When the tumor gradually grows larger and is accompanied by cystic deformation, the tumor density appears inconsistent. CT examination can better show its positional relationship with the blood vessels in the neck. Because the position of the vagal nerve in the cervical sheath is behind the internal carotid artery and internal jugular vein, and the vagal schwannoma is formed based on the gradual increase in the vagal nerve, CT examination often shows that the enlarged tumor deforms and displaces the internal carotid artery. The internal jugular vein is externally displaced, but continuity of the internal jugular and external carotid arteries still exists.^[18,19]^ The content of magnetic resonance imaging (MRI) examination is roughly the same as that of CT, but the advantage of MRI is a higher quality of soft tissue imaging, and for neurogenic tumors, MRI is reported to be able to explore the source of nerves (NOO).^[7]^ In the T1-weighted image, it appears as an iso-density signal, which is similar to the surrounding skeletal muscle, while in the T2-weighted image, it appears as an unevenly high signal due to the characteristics of cell composition and water content.^[6]^

In this case, because of the abnormally abundant blood supply, the continuity of the external carotid artery can be seen on the CT image, indicating that the external carotid artery is not only compressed and deformed but also has branches inserted into the tumor area for blood supply. This phenomenon has rarely been reported in literature. In this case, CT examination was the first choice, and the tumor size, boundary, and positional relationship with the blood vessel were clarified; hence, MRI examination was not performed. DSA was then performed to fully assess the abnormal blood supply, reduce intra-operative bleeding, and keep the surgical field clear.^[15]^ Therefore, for neurogenic tumors with thickened blood vessels on CT images before surgery, although the tumor may be solid, DSA is required for precise diagnosis and design of the operational plan.

Two major methods are used by surgeons in the literature. One was total resection, where the tumor was completely removed, and the perineurium nerves connected to the tumor were sacrificed at the same time. Postoperative complications such as hoarseness, coughing, dysphagia, dysphonia, Horner syndrome, and facial paralysis are more common in patients.^[11]^

Another is subtotal resection, in which the tumor capsule related to the perineurium is preserved and only the core part of the tumor is removed. It can also be called intracapsular resection, a relatively conservative treatment. Owing to the preservation of nerves after surgery, complications are relatively few and mild, and most of them can be recovered.^[10]^ Based on a review of a large amount of previous data, it was found that although the intracapsular excision procedure retains part of the tumor tissue, the postoperative neurological function preservation rate was 60%, and there was no recurrence of the tumor within 1 year which may be related to the inert growth pattern of the tumor. Therefore, intracapsular excision was the primary surgical method used in this case.

Despite improvements in surgical procedures and efforts to prevent postoperative complications, observation is still considered as the first choice for treatment at the early stage because small schwannomas often have no obvious clinical symptoms, which may be discovered by other inspections.^[11]^ The indolent growth and postoperative neurological deficits can cause many complications. Therefore, it is very important to balance the benefits of surgery with the potential harm to postoperative complications.^[21]^ At the same time, patient wishes are also necessary. The risks of surgery and potential lifelong and irreversible complications after surgery must be fully explained to the patient, and an appropriate treatment model should be developed based on the patient's own will.

### 3.2 Reflection on the present case

The present case is a unique case of vagal schwannoma involving a dominant arterial supply. According to previous literature, the fine-needle aspiration biopsy cytology test is often used to determine the nature of the tumor; however, the specificity of the fine-needle aspiration biopsy cytology test is low.^[20,21]^ Hence, in this case, a biopsy under general anesthesia was conducted to confirm the diagnosis. In fact, biopsy by incision can clarify the nature of the tumor and its relationship with the nerves while exploring the vascular condition. Since the amount of bleeding during the biopsy was large enough to raise concerns, DSA examination and embolization therapy were performed. In the present case, we believe that even if it is clearly a solid tumor, as long as the pre-operative CT or MRI examination reveals thick and tortuous blood vessels, the possibility of receiving blood supply from the surrounding arteries should be considered. Although an abnormal arterial blood supply in solid tumors has rarely been reported in the literature, we suggest that DSA should be performed to determine whether there are abnormalities in well-known blood vessels.^[15]^

Vascular embolization is a necessary step before resection of tumor tissues with an abnormal arterial blood supply. It not only reduces the blood supply to the tumor but also keeps the operation field clear. Conventional embolic agents include onyx glue, coils, and gelatin sponge particles. The onyx glue was chosen for this operation because of its advantage in fully blocking the blood supply from the artery to the tumor area, while avoiding blocking the blood supply at the distal end of the external carotid artery branch and retaining the distal blood supply, especially the blood to the brain.^[15]^

## 4 Conclusion

The present paper conducted a short literature review on the characteristics and treatment of schwannomas and reported a unique case that has abundant blood supply and forms malformed vascular nests. The fact that the tumor had an arterial blood supply biopsy was discovered during the biopsy under general anesthesia because of the massive amount of bleeding during the procedure. After the diagnosis was confirmed by DSA, embolization therapy was applied, and subtotal resection of the tumor was performed. A 31-year-old woman recovered well after surgery and showed no significant postoperative complications. Based on the present case, we recommend routine DSA examination before surgery for large tumors in the pharyngeal space, and embolization therapy should be applied if abnormal blood supply is involved. In the future, surgeons and researchers could look into the reason for abnormal blood supply or schwannoma and thus strengthen the understanding of tumors in the pharyngeal area.

## Author contributions

Zheng, Li, and Xu conceived the study. Tao Xu wrote the manuscript. Yue Liu participated in the literature search. Lei Zheng was involved in manuscript revision. All authors have read and approved the final manuscript.

**Data curation:** Yue Liu.

**Formal analysis:** Lei Zheng.

**Project administration:** Siyuan Li, Lei Zheng.

**Resources:** Tao Xu, Yue Liu, Siyuan Li.

**Supervision:** Lei Zheng.

**Writing – original draft:** Tao Xu.

**Writing – review & editing:** Lei Zheng.
